# Suicidal ideations and coping strategies of mothers living with physical disabilities: a qualitative exploratory study in Ghana

**DOI:** 10.1186/s12888-018-1938-x

**Published:** 2018-11-09

**Authors:** Angela Kwartemaa Acheampong, Lydia Aziato

**Affiliations:** 1 School of Nursing, Wisconsin International University College-Ghana, P.O. Box LG 751, Legon, Accra, Ghana; 2 School of Nursing and Midwifery, College of Health Sciences, University of Ghana, P.O. Box LG 43, Legon, Accra, Ghana

**Keywords:** Ghana, Suicide, Mothers with disability, Coping with suicide, Suicidal ideations, Suicidal thoughts and suicidal thoughts

## Abstract

**Background:**

Suicide is higher among adults with disabilities compared to their counterparts without disabilities and suicide is mostly preceded with suicidal ideations. For each successful suicide, there could be many ideations and attempts. Limited scientific data exist on the issue of mothers with disabilities and suicidal ideations. Therefore, this study explored suicidal ideations and coping strategies of mothers living with physical disabilities in Ghana.

**Methods:**

Qualitative descriptive exploratory design was used and data was collected through individualized in-depth interviews. All participants were purposefully selected after informed consent was sought. Data was audiotaped, transcribed and analyzed inductively using content analysis technique.

**Results:**

Almost all the women in this study reported suicidal ideations from time to time. Poisoning was the most predominant means which the women had considered to use if they ever attempted the suicide. Suicidal thoughts were precipitated mainly by discrimination. Their resilience and ability to cope were due to self-motivation, children, counselling, assistance from relatives and prayer.

**Conclusion:**

We concluded that, it is crucial for all health professionals to explore and identify suicidal intentions among mothers with disabilities for them to be referred to the appropriate units for necessary help.

## Background

Suicidal ideation refers to any self-reported thoughts and ideas that involves suicide related conduct which may or in many circumstances may not eventually result in actual suicide [1, 2]. Suicide is still a significant public health problem [3]. The World Health Organization (WHO) estimates that, 800,000 persons commit suicide universally every year which translates into one death every forty seconds [4]. Majority of global suicides (78%) occur in middle and low income countries [5] but most of the suicides that occur in such countries are under reported. Suicide is the second leading cause of death in the world among people between the ages of 15 to 29 years [4].

In Africa, it has been estimated that, 34,000 suicides occur in a year and that translates into 3.2 deaths per 100,000 deaths [6]. Some individuals perceive suicide as acceptable in a situation where there is a disability [7]. Adults with disabilities have higher rates of suicidal ideations compared to those without disabilities [8, 9]. For each successful suicide, there could be many ideations and attempts. It is approximated that, for every successful suicide, there could be twenty attempts and even more ideations [10]. Although there is paucity of literature on suicide in the Ghanaian context, studies have reported fatal and nonfatal suicides among females in Ghana [11, 12].

Depression has been found to have influence on suicide ideations and attempts [13–16]. Mothers with disabilities have a higher probability of developing postpartum depression compared with their counterparts without any disabilities [17]. There is a significant correlation between depression, self-esteem and suicide ideations among people living with disabilities in Ghana and physical disability significantly influenced negative self-esteem [18]. Suicidal tendencies can also be influenced by anxiety and stressful situations. Meanwhile, mothering with physical disabilities can be stressful. Verbal and physical abuse has been associated with suicidal ideations [19, 20] and women with physical disabilities in Ghana have been documented to be physically, socially and verbally abused [21]. Issues about suicide among people living with disabilities could easily be overlooked in a country like Ghana where people living with disabilities are stigmatized [22, 23] coupled with the fact that, attempted suicide is considered a shame [24] and a crime [25]. In addition, some members of professions like psychologists, nurses and police officers support the law that criminalizes suicide in the Ghanaian context [26]. The above reasons could explain why the issues about suicide are not socially discussed in the Ghanaian context. But attempted suicide survivors and their families in Ghana tend to find coping strategies such as social support and personalized spiritual doctrines helpful [24, 27].

There is limited empirical evidence on suicidal ideations among mothers with physical disabilities in Ghana. Therefore, this study sought to explore suicide ideations among mothers with physical disabilities in Ghana. In this study, disability is defined as any physical impairment of the limbs of mothers which causes inefficient use of such limbs leading to interference of activities of daily leading.

## Methodology

### Aim, design and setting

Qualitative descriptive exploratory design was used to gain detailed understanding into the issue of suicidal ideations among mothers with physical disabilities in Ghana. The design was suitable for the problem under investigation since it gave the researcher the chance to probe into participants’ perspectives for themes to emerge. Thus the study aimed to explore the experiences of suicide ideations among mothers with physical disabilities. The study location was a major rehabilitation centre for people living with various forms of disabilities in the Greater Accra Region of Ghana. Participants were drawn from diverse backgrounds in Ghana since the centre accommodates people living with disabilities from the all the ten Regions. The centre is the main rehabilitation facility in the country where people with disabilities across different ethnic groups meet for monthly meetings. Consequently, the context of the study involved mothers with physical disabilities from all parts of Ghana. This brought diversity since participants belonged to diverse ethnic groups of the ten regions of Ghana although residents of the Greater Accra Region. The national disability prevalence is 3%. and 2.6% of the population of Ghanaians in the Greater Accra Region are people with disabilities with majority being females [28].

### Sampling and data collection procedures

Twelve mothers with physical disabilities were recruited into the study after they had been purposefully sampled by the researcher based on the specified inclusion criteria. Mothers with physical disabilities who had biologically given birth in the last ten years and personally cared for them were included in the study. All the recruited participants could speak English or Twi (main Ghanaian local language) since those were the languages which the researchers could understand. The interview guide which was used to stimulate responses was semi structured. All unclear responses were further explored for lucidity. Examples of some questions asked were: *Can you describe any suicidal ideations you have had in the past? What could have triggered such ideations and how did you cope with them? How have you coped with such ideations?* To avoid the researcher’s own biases, leading questions were avoided during the process of data collection and the participants were interviewed in a non-intimidating environment. The interviews were conducted at a secluded area at the rehabilitation center where there was privacy. Therefore, they poured their hearts out. Each interview lasted between thirty (30) and forty (40) minutes. Interviews were audiotaped and transcribed verbatim from audio to text. Before transcription, data which was collected in the local language were translated into English. Transcripts from Twi interviews were discussed with experts in the Twi Language to ensure that participants’ perspectives were communicated. Data reached saturation on the twelfth participant because at this point, no new information was obtained from participants. The venue and time of the interview were at the convenience of the participant.

### Data analysis

Data collection and analysis were done concurrently following the process of content analysis as defined by Padgett [29]. Meanwhile, because NVivo (version 11) was used to organize and manage the data, the manual aspect of data organization as described by Padgett was not used. To understand participants’ perspectives fully, transcripts were read severally before names and phrases were attached to sentences (coding). The codes attached to sentences and phrases were the ones that captured the meanings of the data. Similar codes were assembled together to form sub-themes and sub-themes were also grouped together for major themes to emerge. The research team then met to discuss the themes and sub-themes that had been generated to ensure that participants’ perspectives had been duly represented. The data was then transported into NVivo software (version 11) for management. The study held that the suicidal intentions and coping strategies of participants were not recorded under duress and therefore participants’ perspectives were kept intact.

### Trustworthiness of the study

Concomitant data collection and analysis ensured that emerging themes were probed in subsequent interviews and this brought out the full meanings of the themes. One interview guide was used to interview all participants. Interviews conducted in Twi (local Ghanaian language) were given to experts in that language to review so that transcripts were not misinterpreted. This ensured accuracy of the transcripts. The research team discussed the themes to ascertain that all the data was included in the study. Notes from the field were used to verify and confirm the findings and study processes. Verbatim quotes were used to support the findings and this offered voices to the women.

### Ethical clearance

Ethical clearance was obtained from the Institutional Review Board of the Noguchi Memorial Institute for Medical Research (NMIMR-IRB CPN 038/16–17, FWA 00001824). All the participants gave consents before recruitment into the study. Permission was also sought from participants before interviews were audio-taped. Participants were made aware that, participation in the study was voluntary and withdrawal did not attract any form of penalty. The data presented had no form of identification that could be linked to participants because codes were used to represent participants in the study. The researcher remained non-judgmental and avoided prejudices during data collection.

## Results

### Participants’ background

Twelve (12) participants were recruited into the study. All of them had disabilities pertaining to limb impairments. Therefore, each participant had one or more of her limbs malfunctioning. The women were aged between 21 and 57 years old. Majority of them (10) were Christians while the rest of the two were Muslims. Five of the participants were single while the rest of the seven were living with their partners. All the women were residents at the Greater Accra Region of Ghana and had at least one biological child. Although six of the women were employed, their employment was in the informal sector while the rest were unemployed.

Two major themes emerged after inductive analysis of findings and they included: suicidal ideations and coping strategies. Sub-themes which were aggregated for major themes to emerge were; suicidal thoughts, precipitating factors for suicidal ideations, possible means for suicide execution, hope in God, children, counselling, assistance from relatives, assertiveness, prayer and self-motivation.

### Suicidal ideations

This theme describes the suicidal thoughts, intentions and ideas which participants have ever experienced. It also includes the possible means for committing suicide and the precipitating factors for suicidal ideations.

### Suicidal thoughts

Ten out of the twelve women sometimes had thoughts that made them feel like killing themselves. Some were of the view that, the thoughts occurred at different times of their lives. One of them had more frequent suicidal thoughts when she was a teenager. Some of the women said their suicidal thoughts were prolong and could engage them for a long period. Suicidal thoughts also made the women uncomfortable, disorganized and restless.

***DM11*** *: Sometimes, I feel like killing myself. The thoughts engage me for a long time especially when I happen to go through some circumstances.* ***DM9*** *: Yes, I used to feel like committing suicide. I could have feelings that could last for a loooong time. Anytime I get those thoughts, I became restless.* ***DM8*** *: My suicidal thoughts occurred more often when I was a teenager. It made me very uncomfortable and disorganized.* ***DM5*** *- as for me, I made up my mind to kill myself.*

Two of the women mentioned that, they had never had any suicidal thoughts. One of them was of the view that, she grew up knowing that she was handicapped. Therefore, she had learnt to cope with her circumstances. She narrated:

***DM12*** *: As for that one, I have never had any suicidal ideations before although I was also the only person in my home town who had a physical disability because I have always known myself to be like this so I have learnt to cope with it.*

### Precipitating factors for suicidal thoughts

The women narrated different precipitating factors that normally led to such thoughts. The feeling of discrimination triggered some of the women’s suicidal thoughts. One woman said her siblings used to call her ‘useless’ and that nothing good could come out of her. Such utterances that made her feel neglected were what fueled her need to kill herself. Her narrations are below:

***DM8*** *: When I used to play with my sister, she would make comments like: “You are useless, nothing good would come out of you” It used to bother me when my sister used to say those words to me when I was a child and those were the days I used to have suicidal ideations so that I would die and leave them since I was told that I was useless.*

The discrimination could also emanate from the biological parents of the women. One woman narrated how her feelings of sadness were as a result of rejection from her parents. Some of them were so sad about such treatments to an extent that, they sometimes isolate themselves and cried. Others asked God questions as to why they were the only ones with disabilities in their families. Some women felt disrespected by their relatives and they connected that to be as of the result of their disabilities. Such disrespectful attitudes were what caused their suicidal ideations

***DM5****: When I look at all my siblings, I was the first born and considering how my mother rejected me and then my father also rejected me and then it was only my grandmother who catered for me and my grandmother too wasn’t somebody who had strength, and was also bowlegged… So, sometimes, I could sit somewhere, very quiet and then I could cry so much. And I said, oh God, why is it that only me… in this big family of mine, why is it that only me I have become handicapped and I don’t have help from anywhere? Even now, I can sometimes become very sad and depressed. That even when I am sleeping, I get up from sleep, and then cry just like that on the bed.* ***DM2****: they even respect the abled siblings more than me. As for me, they see me as nothing. When it comes to the dirty works, they will give it to me. That is what makes me sad and makes me have thoughts of killing myself*. ***DM3****: Even my own mother does not give me the necessary respect as the eldest child. Up to date, my younger brother sees himself as an older brother because I am physically challenged. Nobody sees me as the eldest. That makes me sad and makes me want to commit suicide.*

Some of the discriminating attitudes were stemming from the relatives of their spouses. Relatives of the spouses they were married to disapproved of the relationships because they were disabled. Nine of the women reported that, the relatives of their spouses were hostile towards them and could not fathom why women with disabilities were being brought into their families. Some women were not comfortable with the treatment meted out to them by the relatives of their spouses and that was what was triggering their suicidal tendencies. Poor relationships with relatives of their spouses were triggering suicidal thoughts as narrated below:

***DM6*** *: I had suicidal tendencies when I got married. This was because of the way the man and his relatives treated me in those days. The man who impregnated me behaved very well at the beginning of the relationship. All my woes started when I got pregnant and he informed his relatives about it. His relatives rejected me and told him that they were disappointed in him for impregnating a disabled woman* ***DM9*** *: Moreover, I am not too comfortable in my present relationship with my spouse. The relatives of my partner have been worrying me. They disapprove of my relationship with him. When it happens like that, I feel like killing myself and dying out of this world.*

### Possible means for committing suicide

The most predominant means suggested by the women was poisoning. Seven of them had ever thought of swallowing poisons to end their lives while the rest of the three who have ever contemplated suicide thought of hanging themselves from a rope. One of them had attempted to kill herself by means of poisoning. She swallowed the poison and slept off hoping to sleep forever and not to wake up again. Fortunately for her, she woke up without much harm to her apart from the feeling of dizziness.

***DM9*** *: Sometimes, I feel like poisoning myself so that I would die* ***DM3*** *: I tried doing it with poison. I went to buy some poison. It was even meant for rats like, so I mixed it some medicines and I took it. It was. I was feeling dizzy, my eyes was going like this (demonstrating dizziness with her eye balls) so I said oh then bye-bye. I locked my. My mum had gone to church so I locked myself up. I slept off. My mum came, opened the door and called me several times. I said ah mum, am I dead?*

### Coping strategies

Although the women sometimes had suicidal thoughts, they found different ways of coping with it and those were the reason why they were still alive. The coping strategies were geared towards encouraging themselves in order to stay alive. Different coping strategies were adopted. Some encouraged themselves to live for the sake of their children. Others too decided to stay alive and not to commit the actual act of suicide because of counselling, assistance from relatives, prayer and self-motivation.

### Children

Children offered solace to mothers living with physical disabilities. The thought of dying and leaving behind an innocent child was what kept most of the mothers alive. All the mothers could not envisage any other person taking care of the children in their absence. One of the women narrated that, her child is the one who picks the assistive device which she uses as a walking aid for her anytime she attempted to step out and that was what gave her the strength to live.

***DM9*** *: When it happens like that, I ask myself that “who will take care of my children when I die?” but for the sake of my children who are innocent, I choose not to die since these days, nobody takes care of another person’s children. I am living because of my children.* ***DM6*** *: yes, my child gives me hope and gives me a reason to live. When I am about to go out and my child notices it, he quickly goes and brings my crutches to me so that I can move out. He gives me so much hope.* ***DM3*** *: what is keeping me alive is my children. They are the most important thing to me.*

One of the women reported that, her child hired a maid to take care of her before leaving the house and that has kept her alive.

***DM11*** *: before my child left the house, he hired a maid for me so that she would assist me in activities of daily living. She is the reason why I am still alive.*

### Counselling

Counselling received from nurses and religious leaders were recipes for staying alive by some of the women. Nurses counselled them against stressing themselves and thinking about their disabilities. Others had counselling from the leaders of their faith. Such counsel from fellow Christians were so encouraging to an extent that, it kept them from harming themselves.

***DM1*** *: I was thinking about it, but the nurses counselled me that, if I want my baby to be fine I don’t have to think about certain things because if I think about it, it will affect him. So it got to a point that I said maybe that is how God wants it to happen so I took it like that.* ***DM8*** *: At the time I used to have suicidal ideations, I was not a Christian and I was not going to church. When I started going to church and started listening to the words in the Bible, the church members used to counsel me together with other young girls. That was when I told myself that “nooo, I will not kill myself because me too, I am somebody and I would also be an important person in the future.”*

Five mothers had peer counselling from fellow mothers living with disabilities. Peer counselling also encouraged some of them to cope with their suicidal thoughts. The peer counselling were received informally though the sharing of experiences by fellow mothers.

***DM4*** *- when we get together, the counselling from fellow mothers living with disability is so helpful. Those who have older children share their experiences on how they were able to cope and that encourages me a lot.*

### Support from relatives

Six of the mothers with disabilities were fortunate to have some relatives who were willing to support them cope with activities of daily living and that reduced their stress to a large extent. Four of them mentioned their mothers as the ones who had assisted them throughout their lifetime. Two mentioned their sisters as the safe haven in who assisted them to reduce their depression.

***DM11*** *: I have my mother with me whom I consider my all. My mother is like my husband, my sibling, confidant and my everything. My mother is the one who has helped me cope with everything* ***DM6*** *- One of my sisters, had pity on me so she came to stay with me for some time. Her company really reduced my stress and depression.*

### Prayer

Almost all the women (eleven) mentioned prayer as a source of encouragement. They attributed their being alive to prayers. Most of them prayed to God to free them from the suicidal thoughts. To them, if they were freed from such thought, it would give them the peace of mind to live without any suicidal thoughts.

***DM11*** *: My prayer is what is keeping me alive and preventing me from committing suicide. My prayer is that, God should give me strength because as I am growing older, I can feel that my strength is failing me gradually.* ***DM10*** *: I pray fervently that, the Lord would take suicidal thoughts from my head so that I can have my peace of mind to take care of my children once I am still alive.* ***DM5*** *- Still being alive is the work of prayers. I have been praying to God for mercy so that he will forgive me and stop me from having suicidal thoughts. It is my fervent prayer to be free from these thoughts.*

### Self-motivation

All the women in this study motivated themselves sometimes without waiting for any external interventions. Although physically challenged, some of them never felt like they had any form of disabilities. They said that they could do more compared with people who were not physically challenged.

***DM2*** *: because err even me I. I always tell them, as for me, I motivate myself all the time. Although I am physically challenged, I don’t see myself as a disabled because what you can do, I can also do it.* ***DM6*** *: For some time now, I keep telling myself that it would be very stupid if I should kill myself. I tell myself encouraging stuff and that is what has been keeping me alive.*

One of them motivated and disciplined herself to learn a skill when she had the opportunity and that kept her busy. With such a focus, she decided never to despair again and that is how she coped with her suicidal thoughts.

***DM3*** *: I was able to learn to be a professional seamstress for free when I joined this organization. We once went for a national meeting for the physically challenged at Cape Coast and I learnt a great lesson. After seeing other physically challenged individuals, I concluded that I was better off because a lot of persons with disabilities are worse than me. I am far better off. That was when I decided never to be sad and have suicidal thoughts again about my disability.*

## Discussion

This study explored suicidal ideations among mothers living with physical disabilities in the Greater Accra Region of Ghana which highlighted the commonality of the issue among this category of mothers. This is similar to other findings since adults with disabilities have been found to have higher rates of suicidal thoughts compared to their counterparts who have no disabilities [8, 9]. Perhaps disability poses challenges to those who live with it and such challenges may predispose them to depression and subsequent suicidal thoughts. This means that persons living with disabilities should be given special assistance by both the government and relatives.

The women reported discrimination from different angles of their social circles as the main precipitating factor that causes them to have suicidal thoughts. In a similar studies involving adults and trans gender persons, discrimination was reported as the triggering factor that led to suicidal ideations [30, 31]. In a related study, being young and disabled predisposed students to suicidal ideations [32]. Perhaps discriminations makes people living with disability feel isolated. Therefore, that may lead to a sense of separation from the masses and separation may mean committing suicide and leaving this world. Public education should be intensified to emphasize the need to accommodate all manner of persons regardless of their circumstances. Furthermore, laws guiding punishment for those who discriminate against people living with disabilities should be implemented. This may act as a deterrent to people who discriminate against persons with disabilities.

The women mentioned poisoning as the possible means of committing suicide anytime they had suicidal thoughts. In a study in India on a similar subject but in the general population, it was found that, poisoning was the commonest means for committing suicide [33]. Perhaps, poisoning is perceived to be a slow and less violent means of committing suicide and that may be the reason why majority of the women were inclined towards that means. Poisonous substances should not be made readily available to the general public. There should be laws enacted that would regulate the sale of poisonous substances.

Different means of coping mechanisms were adopted by the women to deal with their suicidal thoughts and they included children, counselling, support from relatives, prayer and self-motivation.

Children represent continuity and hope. Some of the women mentioned the fact that their children were the reason why they were still alive. Maybe, the maternal instinct so powerful to an extent that most mothers have special places in their hearts for their children and would therefore want to protect them. Committing suicide and leaving them may expose them to unforeseen dangers. Inferring from this, women with physical disability should be encouraged to procreate so that it would give them a reason to live.

Counselling from nurses and religious leaders allowed the women to cope with their suicidal ideations as reported similarly in other findings [34]. Maybe, counselling gave them information to make wise choices. This calls for the development of strong clinical psychologist units within our hospitals to deal with the psychosocial aspects of disease conditions.

Religion has been drawn on as coping strategy for other groups with suicidal intentions [35]. Prayer as a means of communication with one’s God was perceived by most of the women as their coping mechanism. Prayer was believed to connect them to their God who consoled them and gave them a sense of hope. This coping mechanism is similar to findings of another study since prayer was documented to be used as coping mechanism by suicidal persons in Ghana [27]. Possibly, the thought of having a supreme being gave the women the assurance to live. Counselling sessions should be tailored in such a way that, the God factor should be centralized in bringing hope to women with suicidal ideations.

Mothers with physical disabilities may encounter some difficulties in their quest to meet the demands of motherhood. Therefore, support from relatives is key to the survival and daily upkeep of such women. This was echoed by suicide attempters as a coping strategy in a study in Ghana [27]. Perhaps suicide is a bio-psycho-social problem and the desire to live is influenced positively by healthy social interactions. This suggests that, the general public should be sensitized on the need to lend helping hands to friends and relatives who may be found in such situations.

Self-motivation has been documented to have a correlation with one’s ability to perform a task in some studies [36–38]. The women in this study mentioned self-motivation as a coping strategy to withstand their suicidal thoughts. This may be due to the fact that, personal self-motivation is much more reliant and powerful compared to situations whereby someone else motivates. This suggests that, peer counselling can be scheduled for women in this category for them to share their personal experiences of resilience so that they can learn self-motivation from each other.

The study was limited to mothers with physical limb disabilities. Therefore, the suicidal ideations of mothers with other forms of disabilities such as sight and intellect were not explored. The findings of the study cannot be generalized because of its explorative nature which limits it to small sample size. Further studies on suicidal thoughts of people living with other forms of disabilities in Ghana using both qualitative and quantitative approaches can throw more light on this important public health subject.

## Conclusion

This study gained extensive understanding on the existence of suicidal ideations among mothers living with disabilities in Ghana. Therefore, this study re-emphasized the need for health professionals to always consider all clients as bio-psychosocial beings and therefore the need to treat all aspects of their health. Midwives should support and encourage mothers with disabilities to be resilient. They should always look out for signs of suicidal intentions among mothers with disabilities so that help can be sought for them.

## Abbreviations

WHO: World Health Organization
